# Gleanings in Science and Medicine

**Published:** 1893-05-20

**Authors:** 


### GLEANINGS IN SCIENCE AND MEDICINE.

THE demand for patent medicines in England is greatly in excess of that of other countries, a fact which is probably due to the British law, which places small restrictions on the supply of these various quack compounds. In Russia the case is not parallel, for from those territories all patent medicines are excluded, unless, as is the case with a few exceptions, special permission is first obtained from the Medical Department of the Minister of the Interior. In Sweden, Switzerland, and Turkey the regulations are rather less stringent on this question, but in Germany all proprietary medicines can only be sold by vendors who are legally held responsible for the effect of the drug upon the patient. Much encouragement is not therefore given to the originator of such compounds, especially as all public advertisement of medicines made by secret formula is prohibited. The French laws show little less restriction on the sale of patented nostrums. In England, as we have said, great licence is allowed on this head; indeed, the income of the State derived from this source is at the somewhat high figure of £247,250 annually, which gives us some idea of the immense extent of the consumption of patent medicines in this country.

WHAT have diamond merchants to say to the rivalry now threatened against their trade, and which eventually will probably absorb it? Henri Moisson, at one of the late sittings of the Paris Academy of Sciences, affirmed that he has not only theoretically discovered, but has practically proved that these precious gems can be artificially produced. The process, which he described, is one of an involved technique; but, speaking broadly, it is based upon the actual absorption of carbon by iron at an extremely high temperature, this carbon being again given back in the form of crystals when the iron mass is once more cool and has sufficiently re-hardened. This discovery has an importance outside of the single fact, as it is the outcome, and probably the forerunner, of a continued and laborious research after a closer intimacy with the structure of minerals. In one sense Monsieur Moisson has been anticipated in this the latest work of his laboratory; some time ago two Englishmen, through a different process, arrived at the production of carbonados or black diamonds. These of the French discoverer are, however, of a crystalline structure, having all the properties of the gems with which we have been acquainted. Though the actual size of the diamonds thus produced is, so far, in inverse ratio to the time and labour expended upon them, yet the way is plain and certain, and a more perfect development can be confidently anticipated.

THE Chicago Exhibition has indirectly been the means of giving a fresh impetus to ship-building, and the monster "Campania," the Cunard liner of 30,000 horse-power is the outcome of this. In her construction the latest science of the day has been brought to bear, and she has proved herself to be the fastest passenger steamer on the great Atlantic ferry. Steam was first experimented with in the form of a toy many centuries ago by Hiero of Alexandria. When this idea, of which we now witness such gigantic strides, was first mooted at the Paris Academy of Sciences, Napoleon received it in no complimentary terms. "It is an insane idea," he said, "a gross blunder." Such receptions have not infrequently ushered in the great discoveries of the age. Scientists scoffed and the Church denounced. But though the birth of the steamship was attended by curses, she slowly evolved herself, and in the greater perfection of her later years has proved herself, indeed, to be one of the most useful inventions of science.

